# Keratinocyte Binding Assay Identifies Anti-Desmosomal Pemphigus Antibodies Where Other Tests Are Negative

**DOI:** 10.3389/fimmu.2018.00839

**Published:** 2018-04-24

**Authors:** Federica Giurdanella, Albertine M. Nijenhuis, Gilles F. H. Diercks, Marcel F. Jonkman, Hendri H. Pas

**Affiliations:** Department of Dermatology, University of Groningen, University Medical Center Groningen, Center for Blistering Diseases, Groningen, Netherlands

**Keywords:** pemphigus, diagnosis, autoantibodies, desmosomes, desmogleins, keratinocyte

## Abstract

The serological diagnosis of pemphigus relies on the detection of IgG autoantibodies directed against the epithelial cell surface by indirect immunofluorescence (IIF) on monkey esophagus and against desmoglein 1 (Dsg1) and Dsg3 by ELISA. Although being highly sensitive and specific tools, discrepancies can occur. It is not uncommon that sera testing positive by ELISA give a negative result by IIF and *vice versa*. This brings diagnostic challenges wherein pemphigus has to be ascertained or ruled out, especially when no biopsy is available. We utilized the ability of anti-Dsg3 and anti-Dsg1 IgG to bind in specific desmosomal patterns to living cells to investigate these discrepancies between IIF and ELISA. Living cultured primary normal human keratinocytes were grown under differentiating conditions to induce adequate expression of Dsg1 and Dsg3, incubated with patient serum for 1 h, and then stained to visualize bound IgG. We investigated two different groups; sera from patients with a positive direct immunofluorescence (DIF) and inconsistent serological findings (*n* = 43) and sera with positive ELISA or IIF but with negative DIF (*n* = 60). As positive controls we used 50 sera from patients who fulfilled all diagnostics criteria, and 10 sera from normal human subjects served as negative controls. In the DIF positive group, IgG from 39 of the 43 sera bound to the cells in a desmosomal pattern while in the DIF negative group none of the 60 sera bound to the cells. This shows that for pemphigus patients, ELISA and IIF can be negative while anti-desmosomal antibodies are present and *vice versa* that ELISA and IIF can be positive in non-pemphigus cases. In absence of a biopsy for DIF, such findings may lead to misdiagnosis.

## Introduction

The autoimmune bullous disease pemphigus is caused by the loss of cell–cell adhesion between keratinocytes, induced by autoantibodies mainly directed against the desmosomal cadherins desmoglein 1 (Dsg1) and/or 3 (Dsg3). The serological diagnosis of pemphigus relies on the demonstration of circulating anti-Dsg1 and anti-Dsg3 by ELISA and/or by indirect immunofluorescence (IIF) on monkey esophagus substrate (1). In the pemphigus foliaceus (PF) subgroup, only antibodies against Dsg1 are present, whereas in pemphigus vulgaris (PV) antibodies against Dsg3 are found in mucosal dominant PV (mdPV), or together with anti-Dsg1 in mucocutaneous PV (mcPV) (2). Apart from antibodies to Dsg 1 and 3 also antibodies to desmocollins (Dsc) 1, 2, and 3 are found, but these are more prevalent in atypical pemphigus diseases as paraneoplastic pemphigus, pemphigus herpetiformis, and pemphigus vegetans but are only found sporadically in classical PV and PF (3, 4). In addition to cadherins, dozens of other proteins were shown to be recognized by pemphigus IgG, including muscarinic and nicotinic acetylcholine receptors, the neonatal Fc receptor, and mitochondrial proteins but proof that these non-desmosomal antibodies have a role in acantholysis is lacking (5). Although being a highly sensitive and specific tool, the ELISA for Dsg1 and Dsg3 ectodomains can be positive without clinical or other laboratory evidence for actual pemphigus, and a number of reports documenting a lack of concordance between positive ELISA and the final diagnosis of the patient can be found in the literature (6). Also, we recently reported the possibility of discrepancies for biopsy proven pemphigus patients, where we found that 9% of positive ELISA sera tested negative by IIF, while 6% of IIF positive sera were negative by ELISA and 5% of pemphigus patient had completely negative serology (7). The latter can be due to a low level of antibodies that is not detectable by IIF or ELISA, to the cessation of antibody production while the skin is still loaded with IgG or that the disease is driven by non-desmosomal antibodies. Inconsistent findings during the diagnostic process bring decision-making challenges wherein the diagnosis of pemphigus needs to be confirmed or ruled out, especially when no biopsy is available. Here, we employed as a new method the ability of pemphigus serum IgG to bind to living cultured keratinocytes to understand discrepancies in currently used diagnostic serum assays.

## Materials and Methods

### Human Sera

**Figure 1:**
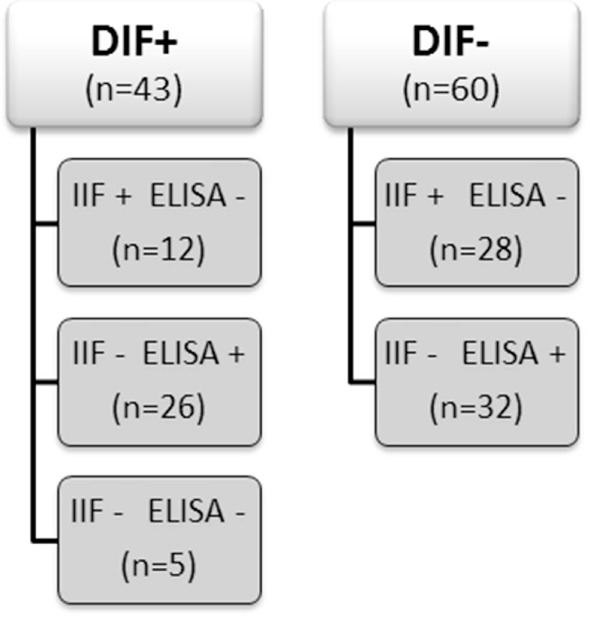
Groups’ characteristics and sampling number.

From our database of the Center for Blistering Diseases in the Netherlands, we retrospectively selected sera from patients that had pemphigus in the differential diagnosis and at least one positive test, direct immunofluorescence (DIF), IIF on monkey esophagus, or Dsg ELISA. This retrospective study with leftover sera from diagnostic tests does not need approval of the ethics committee in the Netherlands. We included 103 sera and divided them in two groups according to the positive or negative result at DIF analysis for IgG deposits at the epithelial cell surface (ECS). Each group was subsequently branched in subgroups according to the results obtained by IIF and by ELISA as routinely performed in the diagnostic work up of autoimmune bullous diseases (Figure 1). As double check, all sera were retested by both IIF on monkey esophagus and, to avoid false positives due to precursor epitopes, also by MBL MESACUP-2 Dsg ELISA. In the DIF negative group, 18 sera were positive for anti-Dsg1 antibodies (median 36.5, Q1 30, Q3 43), and 14 sera were positive for anti-Dsg3 antibodies (median 34.5, Q1 29, Q3 45). Of the 60 DIF negative patients, the final diagnosis was available for 49 patients and included among others 10 cases of lichen planus and 7 cases of pemphigoid but no case of pemphigus (Table 1). For the validation of the *in vitro* assay, we used as positive controls sera from 50 PV and PF patients whose diagnosis had been ascertained by concordance between DIF, IIF, ELISA, and clinical and histopathological findings. Sera from 10 healthy human subjects served as negative controls.

**Table 1:** Final diagnoses of patients in the direct immunofluorescence negative group.

Definite diagnosis	No. of cases
Lichen planus	10
Bullous pemphigoid	7
Pruritus sine materia	4
Aphthosis	3
Eczema	3
Impetigo bullosa	2
Pityriasis rubra pilaris	2
Prurigo nodularis	2
Pseudoporphyria	2
Vasculitis	2
Brachioradial pruritus	1
Dermatitis factitia	1
Erosive pustular scalp dermatosis	1
Genital ulcer	1
Gingivitis	1
Graft versus host disease	1
Lichen planus pemphigoid	1
Lupus erythematosus	1
Mucous membrane pemphigoid	1
Nummular eczema	1
Urticaria	1
Vulvar carcinoma	1

*Final diagnoses were unavailable in 11 sera that were sent from different hospitals for a second opinion*.

### Keratinocyte Binding Assay (KBA)

NHK were isolated from redundant healthy skin obtained from breast reduction surgery upon written informed consent and grown on glass coverslips in 24-well plates using CnT-Prime and CnT-Prime 2D Differentiation medium (CELLnTEC, Switzerland) at 37°C and 5% CO_2_. When shifted to 1.2 mM calcium medium, desmosomes are induced that initially contain Dsg3 but not Dsg1. After prolonged incubation, cells start to differentiate and to synthesize Dsg1. Hence, we used cells 4 days after calcium shift to test binding of patient IgG to desmosomal proteins. NHK on coverslips were incubated with 2.5% serum in culture medium for 1 h at 37°C after which the cells were fixed in 2% formaldehyde and stored frozen at −80°C until staining. Fixed keratinocytes were stained using DyLight488-labeled goat-anti-human IgG and DAPI for the nuclear staining. The KBA was scored positive for anti-Dsg1 antibodies if IgG bound only to large differentiated cells, and positive for anti-Dsg3 antibodies if IgG bound to all cells. If all cells bind IgG then it is impossible to assess the presence of concomitant anti-Dsg1 IgG as the large differentiated cells also express a high level of Dsg3. Hence, the term “not determinable.” The KBA was scored negative if no binding to human IgG was observed. Coverslips were examined under a Leica DMRA fluorescence microscope and images acquired by a Leica DFC350 FX digital camera (Leica, Germany).

### Dsg1 and Dsg3 ELISA

All sera were tested by the MESACUP-2 ELISA test for anti-Dsg1 and anti-Dsg3 antibodies (MBL, Japan). As a second means of comparison of the negative ELISA results of sera that tested positive in the KBA (*n* = 13), the anti-Dsg1 and anti-Dsg3 microplate ELISA (EUROIMMUN AG, Germany) was used. Both tests were performed according to the manufacturers’ respective protocols, and a cutoff value of 20 U/ml was used to define positivity.

## Results

### Keratinocyte Binding Assay

**Figure 2:**
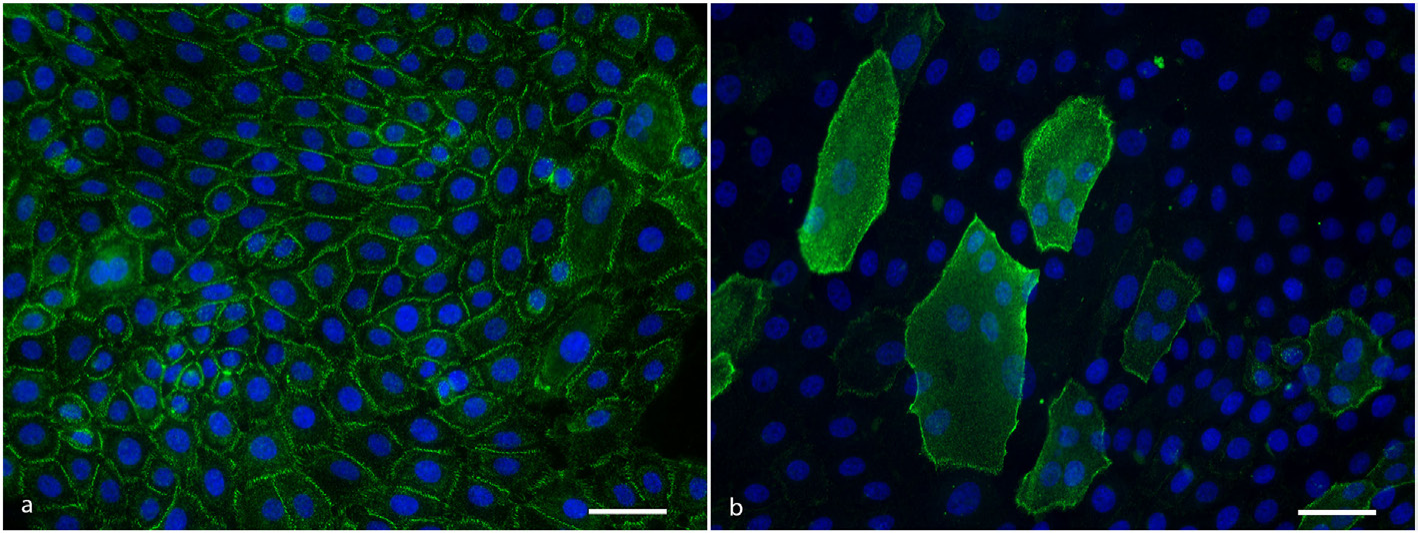
**(A)** Anti-Dsg3 IgG binding pattern to cultured keratinocytes showing desmosomal distribution at cell–cell contacts. Scale bar is 50 µm.**(B)** Anti-desmoglein 1 IgG binding pattern to cultured keratinocytes showing a desmosomal distribution at the cell periphery of differentiated cells. Scale bar is 50 µm.

IgG from anti-Dsg3 containing sera binds to all keratinocytes in a typical desmosomal pattern (Figure 2A). IgG directed against Dsg1 binds in a desmosomal pattern to differentiated cells only that are easily recognizable as they are much larger than undifferentiated cells and lie on top of them (Figure 2B). All PF sera and all PV sera of the positive control group bound to the cells in the expected patterns, and of the 10 normal human sera not one serum bound to the cells.

In the DIF+ groups 91% of the sera reacted with cultured keratinocytes by binding of IgG in a desmosomal pattern. Other patterns were not observed. In the IIF+ group, 10 of the 12 sera tested positive for desmosomal IgG binding (Table S1 in Supplementary Material). In the ELISA+ group, all 26 sera tested positive for desmosomal binding. Interestingly, two sera (Table S1 in Supplementary Material, #30, #32) positive for both anti-Dsg1 and anti-Dsg3 IgG showed binding in the Dsg1 pattern only, which was in line with the clinical presentation of both patients that suggested PF instead of PV. IgG from one serum positive for anti-Dsg1 (Table S1 in Supplementary Material, #34) and one serum positive for anti-Dsg3 (Table S1 in Supplementary Material, #36) bound in a converse pattern to the cells; in the first case, erosive lesions limited to the foreskin were present, while in the second case both skin and oral involvement were noted. In the serologically negative group, two sera bound to the cells in the Dsg1 pattern (Table S1 in Supplementary Material, #39, #42) and in one case in the Dsg3 pattern (Table S1 in Supplementary Material, #43). None of the DIF− sera showed a desmosomal binding to cells. Results are summarized in the Table S2 in Supplementary Material.

### Dsg1 and Dsg3 ELISA

The sera that bound to cells but were negative by routine MBL ELISA were also assayed by a Dsg ELISA kit from another manufacturer. Although one serum tested positive with this kit, the other 12 remained negative. Complete ELISA results are listed in Table 2.

**Table 2:** Alternative ELISA results for sera testing positive for keratinocyte binding but negative on first ELISA.

Group	Sample	MBL ELISA desmoglein 1 (Dsg1)	MBL ELISA Dsg3	EU ELISA Dsg1	EU ELISA Dsg3	KBA Dsg1	KBA Dsg3
D+ I+ E−	1	1	3	2	2	nd	+
D+ I+ E−	2	3	2	2	3	nd	+
D+ I+ E−	3	1	4	2	3	+	−
D+ I+ E−	4	2	17	2	6	nd	+
D+ I+ E−	5	19	13	2	15	nd	+
D+ I+ E−	6	9	5	−14	−4	nd	+
D+ I+ E−	7	8	14	3	8	nd	+
D+ I+ E−	8	3	16	1	10	nd	+
D+ I+ E−	10	6	5	**34**	**45**	nd	+
D+ I+ E−	12	4	1	2	2	nd	+
D+ I−E−	38	17	1	6	2	+	−
D+ I− E−	41	1	0	−11	−7	+	−
D+ I− E−	42	6	2	−10	−3	nd	+

*Values are expressed in U/mL*.

*Positive results are in bold*.

*D, direct immunofluorescence; I, indirect immunofluorescence; E, Dsg ELISA; EU, euroimmun; KBA, keratinocyte binding assay; nd, not determinable*.

## Discussion

Here, we developed a KBA as an additional test based on the ability of pemphigus IgG to bind to desmosomes of living keratinocytes. The KBA discriminates PV from PF but cannot discriminate between mdPV and mcPV as differentiated cells also express Dsg3. Although not qualitative it is a very sensitive assay as in the developing phase of the KBA, we found that when titers decrease and the ELISA turns negative the KBA remains positive (data not shown). Furthermore, similar to ELISA, the assay does not discriminate between pathogenic and non-pathogenic antibodies as sera from patients in remission off-medication with positive anti-Dsg3 titers also bound to the cells (unpublished results).

Our results show that the KBA has a high correlation with the DIF, we tested in total here 93 sera with positive DIF and 97% of the sera bound to the cells in the specific desmosomal patterns (for details see Table 3). This is a higher sensitivity than IIF and ELISA which we recently calculated to be, respectively, 86 and 89% (7). However, in contrast to IIF and ELISA, the KBA is labor intensive and also not quantitative and therefore not a suitable replacement for either IIF or ELISA. Of the 60 sera with negative DIF, none of the sera bound to the cells. As we reported before, a negative DIF is extremely rare in pemphigus and therefore pemphigus is not expected for this group, and indeed the final diagnosis that we could retrieve for 49 of these patients differed from pemphigus.

**Table 3:** Values of sensitivity and specificity calculated for positive and negative controls, biopsy proven pemphigus patients and negative DIF patients groups.

Group	*n*	KBA+	Sensitivity (%)	Specificity (%)
Positive controls	50	50	100	–
D+ I+ E−	12	10	83.3	–
D+ I− E+	26	26	100	–
D+ I− E−	5	3	60	–
NHS	10	0	–	100
D− I+ E−	28	0	–	100
D− I− E+	32	0	–	100

*D, direct immunofluorescence; I, indirect immunofluorescence; E, Dsg ELISA; NHS, normal human subjects; KBA, keratinocyte binding assay*.

Interestingly, in two cases, we observed binding in a converse pattern than the antibodies’ profile determined by ELISA. In the first case (#34), only anti-Dsg1 IgG were detected by ELISA, but lesions were confined to the foreskin and the histopathology showed a suprabasal intraepidermal split, fitting a diagnosis of PV and presence of anti-Dsg3 antibodies as detected by KBA. The second case (#36) was a patient presenting with mucocutaneous involvement but both ELISA and KBA detected a different antibody profile than expected. The reason for this discrepancy is unclear.

A few reports in literature describe the existence of antibodies against Dsg1 and/or Dsg3 in patients affected by skin conditions other than pemphigus especially patients affected by lichen planus. Given explanations vary between these antibodies having an actual role in the pathogenesis of oral lichen planus or that they would be non-pathogenic (8–10). DIF was only performed in one of these studies, and both patients had no ECS IgG deposition (10). Most of the our lichen planus patients had a DIF with fibrin deposition along the BMZ, but no deposition in ECS pattern although in one case some irregular IgG deposition was seen in the epidermis. Four sera displayed anti-ECS antibodies and six were positive in ELISA, either for anti-Dsg1 or anti-Dsg3, or for both anti-Dsg1 and anti-Dsg3. Although positive in ELISA, they did not bind to cells. Seen that in actual active pemphigus, we clearly found binding when ELISAs became negative (values below 9) the question evolves to what these antibodies bind on the ELISA plates. As we used MESACUP-2 ELISA, it cannot be to the precursor form of Dsg. If they are non-pathogenic antibodies, then they differ from non-pathogenic antibodies in pemphigus patients in complete remission off therapy as these do bind to the cells. It could be that the antibodies are directed to a cryptotope that becomes unmasked in recombinant Dsg present on the ELISA plate. A last explanation is that they are just false positives caused by IgG binding to some other non-Dsg component on the plate, although we then would have expected them to be positive in both ELISAs. Interestingly, 10 sera that bound to the cells (KBA+) were IIF positive but were negative in ELISA kits from different manufacturers. Some patients had positive ELISAs at start of the disease but these turned negative during the course of disease, while IIF stayed positive. Other patients never developed a positive ELISA. As the binding patterns were desmosomal the underlying protein must be desmosomal or desmosome associated. Prime candidates are Dsc and seen the binding of nine sera to all cells, Dsc3 would be the prime suspect. However, eight of these sera were included in a previous study on anti-Dsc3 antibodies, and only one of these was positive in an assay on transfected HEK cells that expressed the ectodomain of Dsc3 on the cell surface (4). In this same study, all eight sera tested also negative for anti-Dsc1 and anti-Dsc2 antibodies. Other antigens have been suggested to serve as pemphigus antigens including muscarinic and nicotinic acetylcholine receptors, pemphaxin, and mitochondrial proteins (11–14). But these antigens are non-desmosomal and therefore cannot explain the observed desmosomal binding patterns. Despite being negative in the Dsg3 ELISA, we feel that Dsg3 is still the prime candidate as antigen. It is possible that we are dealing with low titer high avidity anti-Dsg3 antibodies here that have index values below the cutoff of the ELISA. We have observed patients with active disease that during titer monitoring occasionally had ELISA values just above the cutoff value but mostly, under still active disease, under the cutoff value (unpublished results).

For daily practice of diagnosis, our binding assay results indicate that ELISA and IIF should be interpreted with care as they are positive in a considerable number of cases without other laboratory or clinical evidence of pemphigus. We estimated that in our database for the period 1 January 2002 until 1 January 2017, about 13% of patients with positive ELISAs for Dsg did not have pemphigus. Therefore, taking serum but no biopsy for diagnosis, which is a regularly encountered procedure in the consulting room, bears a risk to reach a false conclusion. In our database, we found 32 sera with no concomitant biopsy that tested positive in either IIF or ELISA, while only 2 (6%) were capable of binding to cells in the KBA (Table S3 in Supplementary Material).

The KBA we demonstrate here is extremely sensitive and reliable. For specialized laboratories, it can provide additional information in challenging cases where the diagnosis of pemphigus cannot be decisively confirmed nor ruled out by routine serological analysis. Furthermore, it is an additional research tool to investigate the nature of pemphigus antibodies.

## Author Contributions

FG, HP, and MJ contributed to the design of the study. FG and AN performed the experiments. FG and HP wrote the manuscript. GD and MJ revised the manuscript.

## Conflict of Interest Statement

The authors declare that the research was conducted in the absence of any commercial or financial relationships that could be construed as a potential conflict of interest.
