# Supplementary material for: Keratinocyte Binding Assay Identifies Anti-Desmosomal Pemphigus Antibodies Where Other Tests Are Negative
Source: Front Immunol. 2018 Apr 24;9:839. doi: 10.3389/fimmu.2018.00839 (PMC5928912; doi:10.3389/fimmu.2018.00839)
Supplement: Supplementary file 1 [file Table_1.docx]

Supplementary Material

Keratinocyte binding assay identifies anti-desmosomal pemphigus antibodies where other tests are negative

Federica Giurdanella, Albertine M. Nijenhuis, Gilles F.H. Diercks, Marcel F. Jonkman, Hendri H. Pas^*^

*** Correspondence:** Hendri H Pas: h.h.pas@umcg.nl

**Table S1 -** Comprehensive results for the DIF positive pemphigus sera. ELISA values are expressed in U/mL. IIF-MO: indirect immunofluorescence on monkey esophagus. Positive results are in bold. KBA: keratinocyte binding assay. nd: not determinable.

| **Sample** | **ELISA Dsg1** | **ELISA Dsg3** | **IIF-MO** | **KBA Dsg1** | **KBA Dsg3** |
| --- | --- | --- | --- | --- | --- |
| 1 | 1 | 3 | **+** | nd | **+** |
| 2 | 3 | 2 | **+** | nd | **+** |
| 3 | 1 | 4 | **+** | **+** | - |
| 4 | 2 | 17 | **+** | nd | **+** |
| 5 | 19 | 13 | **+** | nd | **+** |
| 6 | 9 | 5 | **+** | nd | **+** |
| 7 | 8 | 14 | **+** | nd | **+** |
| 8 | 3 | 16 | **+** | nd | **+** |
| 9 | 4 | 2 | **+** | - | - |
| 10 | 6 | 5 | **+** | nd | **+** |
| 11 | 1 | 1 | **+** | - | - |
| 12 | 4 | 1 | **+** | nd | **+** |
| 13 | **96** | 1 | **-** | **+** | - |
| 14 | **52** | 2 | **-** | **+** | - |
| 15 | **>150** | 2 | **-** | **+** | - |
| 16 | **138** | 15 | **-** | **+** | - |
| 17 | **123** | 1 | **-** | **+** | - |
| 18 | **124** | 2 | **-** | **+** | - |
| 19 | **>150** | 5 | **-** | **+** | - |
| 20 | **>150** | 4 | **-** | **+** | - |
| 21 | **113** | 1 | **-** | **+** | - |
| 22 | **71** | 1 | **-** | **+** | - |
| 23 | **110** | 2 | **-** | **+** | - |
| 24 | **72** | 3 | - | + | - |
| 25 | 6 | **84** | **-** | nd | **+** |
| 26 | 6 | **52** | **-** | nd | **+** |
| 27 | 4 | **55** | **-** | nd | **+** |
| 28 | 2 | **37** | **-** | nd | **+** |
| 29 | **134** | **101** | **-** | nd | **+** |
| 30 | **95** | **22** | **-** | **+** | - |
| 31 | **115** | **95** | **-** | nd | **+** |
| 32 | **86** | **26** | **-** | **+** | - |
| 33 | **74** | **79** | **-** | nd | **+** |
| 34 | **24** | 3 | - | **-** | **+** |
| 35 | **44** | 15 | - | **+** | **-** |
| 36 | 8 | **33** | - | **+** | **-** |
| 37 | 2 | **21** | - | nd | **+** |
| 38 | 2 | **37** | - | nd | **+** |
| 39 | 17 | 1 | - | **+** | **-** |
| 40 | 8 | 2 | - | **-** | **-** |
| 41 | 1 | 1 | - | **-** | **-** |
| 42 | 1 | 0 | - | **+** | **-** |
| 43 | 6 | 2 | - | nd | **+** |
