# Supplementary material for: Keratinocyte Binding Assay Identifies Anti-Desmosomal Pemphigus Antibodies Where Other Tests Are Negative
Source: Front Immunol. 2018 Apr 24;9:839. doi: 10.3389/fimmu.2018.00839 (PMC5928912; doi:10.3389/fimmu.2018.00839)
Supplement: Supplementary file 2 [file Table_2.docx]

Supplementary Material

**Keratinocyte binding assay identifies anti-desmosomal pemphigus antibodies where other tests are negative**

Federica Giurdanella, Albertine M. Nijenhuis, Gilles F.H. Diercks, Marcel F. Jonkman, Hendri H. Pas^*^

*** Correspondence:** Hendri H Pas: h.h.pas@umcg.nl

**Table S2 -** Comprehensive results for the DIF negative pemphigus sera. ELISA values are expressed in U/mL. IIF-MO: indirect immunofluorescence on monkey esophagus. Positive results are in bold. KBA: keratinocyte binding assay.

| **Sample** | **ELISA Dsg1** | **ELISA Dsg3** | **IIF-MO** | **KBA Dsg1** | **KBA Dsg3** |
| --- | --- | --- | --- | --- | --- |
| 44 | 1 | 2 | + | - | - |
| 45 | 3 | 2 | + | - | - |
| 46 | 4 | 5 | + | - | - |
| 47 | 0 | 8 | + | - | - |
| 48 | 3 | 3 | + | - | - |
| 49 | 16 | 12 | + | - | - |
| 50 | 1 | 3 | + | - | - |
| 51 | 1 | 1 | + | - | - |
| 52 | 1 | 9 | + | - | - |
| 53 | 4 | 1 | + | - | - |
| 54 | 5 | 2 | + | - | - |
| 55 | 11 | 15 | + | - | - |
| 56 | 3 | 1 | + | - | - |
| 57 | 5 | 1 | + | - | - |
| 58 | 1 | 1 | + | - | - |
| 59 | 2 | 0 | + | - | - |
| 60 | 2 | 2 | + | - | - |
| 61 | 4 | 1 | + | - | - |
| 62 | 2 | 1 | + | - | - |
| 63 | 8 | 1 | + | - | - |
| 64 | 5 | 1 | + | - | - |
| 65 | 1 | 1 | + | - | - |
| 66 | 5 | 3 | + | - | - |
| 67 | 2 | 4 | + | - | - |
| 68 | 1 | 1 | + | - | - |
| 69 | 1 | 0 | + | - | - |
| 70 | 2 | 3 | + | - | - |
| 71 | 1 | 3 | + | - | - |
| 72 | **43** | 1 | - | - | - |
| 73 | **57** | 2 | - | - | - |
| 74 | **61** | 1 | - | - | - |
| 75 | **25** | 2 | - | - | - |
| 76 | **55** | 2 | - | - | - |
| 77 | **30** | 6 | - | - | - |
| 78 | **36** | 5 | - | - | - |
| 79 | **38** | 15 | - | - | - |
| 80 | **32** | 1 | - | - | - |
| 81 | **37** | 0 | - | - | - |
| 82 | **27** | 12 | - | - | - |
| 83 | **22** | 8 | - | - | - |
| 84 | **36** | 1 | - | - | - |
| 85 | **36** | 1 | - | - | - |
| 86 | **26** | 9 | - | - | - |
| 87 | **39** | 19 | - | - | - |
| 88 | **70** | 2 | - | - | - |
| 89 | **42** | 1 | - | - | - |
| 90 | 2 | **22** | - | - | - |
| 91 | 4 | **42** | - | - | - |
| 92 | 1 | **33** | - | - | - |
| 93 | 4 | **45** | - | - | - |
| 94 | 2 | **84** | - | - | - |
| 95 | 14 | **45** | - | - | - |
| 96 | 12 | **53** | - | - | - |
| 97 | 1 | **27** | - | - | - |
| 98 | 11 | **29** | - | - | - |
| 99 | 3 | **29** | - | - | - |
| 100 | 129 | **30** | - | - | - |
| 101 | 33 | **35** | - | - | - |
| 102 | 43 | **34** | - | - | - |
| 103 | 63 | **35** | - | - | - |
