# Supplementary material for: Keratinocyte Binding Assay Identifies Anti-Desmosomal Pemphigus Antibodies Where Other Tests Are Negative
Source: Front Immunol. 2018 Apr 24;9:839. doi: 10.3389/fimmu.2018.00839 (PMC5928912; doi:10.3389/fimmu.2018.00839)
Supplement: Supplementary file 3 [file Table_3.docx]

Supplementary Material

Keratinocyte binding assay identifies anti-desmosomal pemphigus antibodies where other tests are negative

Federica Giurdanella, Albertine M. Nijenhuis, Gilles F.H. Diercks, Marcel F. Jonkman, Hendri H. Pas^*^

*** Correspondence:** Hendri H Pas: h.h.pas@umcg.nl

**Table S3 -** Comprehensive results for 32 serum samples with no biopsy available for DIF analysis. ELISA values are expressed in U/mL. IIF-MO: indirect immunofluorescence on monkey esophagus. Positive results are in bold. KBA: keratinocyte binding assay.

| **Sample** | **ELISA Dsg1** | **ELISA Dsg3** | **IIF-MO** | **KBA Dsg1** | **KBA Dsg3** |
| --- | --- | --- | --- | --- | --- |
| 1 | 1 | 1 | **+** | - | - |
| 2 | 12 | 17 | **+** | - | - |
| 3 | 1 | 1 | **+** | - | - |
| 4 | 13 | 8 | **+** | - | - |
| 5 | 4 | 1 | **+** | - | - |
| 6 | 0 | 1 | **+** | - | - |
| 7 | 2 | 2 | **+** | - | - |
| 8 | 3 | 3 | **+** | - | - |
| 9 | 6 | 1 | **+** | - | - |
| 10 | 1 | 1 | **+** | - | - |
| 11 | 5 | 3 | **+** | - | - |
| 12 | 2 | 6 | **+** | - | - |
| 13 | 4 | 4 | **+** | - | - |
| 14 | 2 | 1 | **+** | - | - |
| 15 | 15 | 10 | **+** | - | - |
| 16 | 3 | 1 | **+** | - | - |
| 17 | 6 | 3 | **+** | - | - |
| 18 | 2 | 1 | **+** | - | - |
| 19 | 3 | 2 | **+** | - | - |
| 20 | 2 | 1 | **+** | - | - |
| 21 | 2 | 1 | **+** | - | - |
| 22 | 1 | 0 | **+** | - | - |
| 23 | 6 | 1 | **+** | **+** | - |
| 24 | 4 | 2 | **+** | - | - |
| 25 | 1 | 1 | **+** | - | - |
| 26 | 1 | 1 | **+** | - | - |
| 27 | 1 | 1 | **+** | - | - |
| 28 | **28** | 0 | - | - | - |
| 29 | **79** | 0 | - | **+** | - |
| 30 | **25** | **15** | - | - | - |
| 31 | 4 | **28** | - | - | - |
| 32 | 3 | **53** | - | - | - |
